# Subdural evacuating port system with subdural thrombolysis for the treatment of chronic subdural hematoma in patients older than 80 years

**DOI:** 10.3389/fneur.2023.1068829

**Published:** 2023-02-15

**Authors:** Tianqing Liu, Zhenwen Gao, Jianjun Zhou, Xiaoyan Lai, Xiaomei Chen, Qiong Rao, Dongbin Guo, Jinliang Zheng, Fuxin Lin, Yuanxiang Lin, Zhiqin Lin

**Affiliations:** ^1^Department of Neurosurgery, Longyan First Affiliated Hospital of Fujian Medical University, Longyan, Fujian, China; ^2^Department of Neurosurgery, The First Affiliated Hospital, Fujian Medical University, Fuzhou, China

**Keywords:** chronic subdural hematoma, surgical treatment, subdural evacuating port system, complication, outcome, recurrence, very elderly patients

## Abstract

**Objective:**

The subdural evacuating port system (SEPS) is a minimally invasive approach that can be performed under local anesthesia for the treatment of chronic subdural hematoma (CSDH). Subdural thrombolysis has been described as an exhaustive drainage strategy and found to be safe and effective for improving drainage. We aim to analyze the effectiveness of SEPS with subdural thrombolysis in patients older than 80 years.

**Method:**

Consecutive patients aged ≥80 years old who presented with symptomatic CSDH and underwent SEPS followed by subdural thrombolysis between January 2014 and February 2021 were retrospectively studied. Outcome measures included complications, mortality, recurrence, and modified Rankin Scale (mRS) scores at discharge and 3 months.

**Results:**

In total, 52 patients with CSDH in 57 hemispheres were operated on, with a mean age of 83.9 ± 3.3 years, and 40 (76.9%) patients were men. The preexisting medical comorbidities were observed in 39 patients (75.0%). Postoperative complications occurred in nine patients (17.3%), with two having significant complications (3.8%). The complications observed included pneumonia (11.5%), acute epidural hematoma (3.8%), and ischemic stroke (3.8%). One patient experienced contralateral malignant middle cerebral artery infarction and died of subsequent severe herniation, contributing to a perioperative mortality rate of 1.9%. Discharge and 3 months of favorable outcomes (mRS score 0–3) were achieved in 86.5% and 92.3% of patients, respectively. CSDH recurrence was observed in five patients (9.6%), and repeat SEPS was performed.

**Conclusion:**

As an exhaustive drainage strategy, SEPS followed by thrombolysis is safe and effective with excellent outcomes among elderly patients. It is a technically easy and less invasive procedure with similar complications, mortality, and recurrence rates compared with burr-hole drainage in the literature.

## Introduction

Chronic subdural hematoma (CSDH) is one of the most common diagnoses encountered by neurosurgeons in clinical practice. The annual incidence of CSDH increases with age, and CSDH is more prevalent in people aged 65 years and older (1). Symptomatic CSDH has a significant impact on the quality of life and overall functionality of geriatric patients. Surgical evacuation is currently the mainstay treatment for symptomatic patients. According to previous reports of CSDH in patients aged 80 years and older, the perioperative morbidity and mortality rates ranged from 4.0 to 37.5 and 3.6 to 8.8%, respectively, with recurrence rates varying between 3.6 and 25.0% (2–8). However, most of these patients were treated by burr-hole drainage (BHD) under general anesthesia.

When compared with BHD, the subdural evacuating port system (SEPS) is a technically less complex and invasive procedure that can be performed at the bedside with local anesthesia alone (9–12). It is theoretically indicated for very elderly patients who have multiple comorbidities and are at increased risk for general anesthesia. Moreover, the role of SEPS in the treatment of CSDH has evolved rapidly, initially as an alternative to traditional BHD but more recently as a first-line procedure (9, 13, 14). A recent large retrospective observational study has shown evidence that the overall performance characteristics of SEPS and BHD were similar (13). Nonetheless, there is a paucity of literature characterizing outcomes in very elderly patients undergoing SEPS.

Subdural thrombolysis has been described as a safe and effective strategy for improving drainage (15–17). The purpose of this study is to analyze the effectiveness of SEPS with subdural thrombolysis for the treatment of CSDH in such a peculiar population for which specific guidelines are not available.

## Methods

### Patient population

Data on consecutive patients aged ≥80 years old who underwent SEPS for CSDH from January 2014 to February 2021 were retrospectively collected. The data analyzed included patients' age, sex, history of head injury, medical history, antithrombotic medication history, Glasgow Coma Scale (GCS), and modified Rankin Scale (mRS) scores at admission, discharge, and 3 months following surgery, neurological symptoms, image findings, and clinical outcomes. The CSDH was classified into four types according to the CT imaging appearance based on density changes: homogeneous, laminar, separated, and trabecular types (18). The study was approved by the Ethics Committee of Longyan First Hospital, Fujian Medical University. All study protocols and procedures were conducted in accordance with the Declaration of Helsinki.

### Surgical procedure

**Figure 1:**
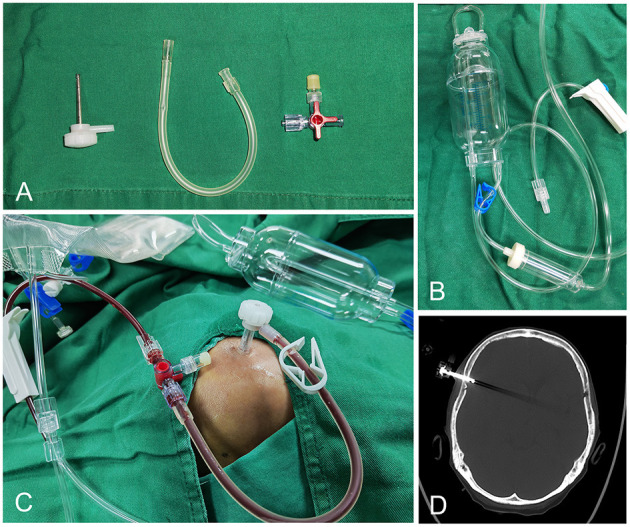
The components of SEPS: Stainless steel evacuating port, silicone tubing, three-way stopcock, and hermetical drainage system **(A, B)**. The evacuating port is connected to the silicone tubing, a three-way stopcock, and a closed drainage system **(C)**. Postoperative CT bone window demonstrates the port in position **(D)**.

The SEPS consists of two systems; a stainless steel evacuating port that can be threaded through the twist-drill hole into the cranial cavity and a hermetical drainage system that is attached to the port to evacuate the hematoma fluid. The SEPS is placed under local anesthesia in the operating room according to standard techniques. The evacuating port was then connected to the silicone tubing, a three-way stopcock, and a closed drainage system (Figure 1).

Regarding postoperative management, the draining system was generally fixed to the bed's frame at the level of the external acoustic meatus in the supine position but sometimes it should be individualized. The patients were limited to bed rest as much as possible in the 15 degrees head-up tilt position. Sitting position or mobilization was allowed if the drainage was shut off. A CT scan was done at 4–6 h following the surgery to confirm the positioning of the port and to assess the adequacy of drainage. Subsequent CT scans were conducted for any safety concerns or repeatedly until the residual hematoma was totally removed or the drainage was clean.

6 h or more after SEPS placement, we administered the thrombolytic agent urokinase directly into the subdural space through a three-way stopcock, at 50,000 IU in 5 mL followed by a 2 mL flush of sterile saline. The drainage was shut off for 1 h to allow drug-hematoma interaction and then reopened. We repeat this administration daily. The SEPS was removed if postoperative imaging demonstrated total evacuation of the hematoma, or the drainage was clean.

The discharged patients were followed for more than 3 months and underwent a routine clinical assessment 1 to 3 months after surgery, including neurological examination and CT scan of the head. The mRS score was determined on the basis of the information obtained.

### Patient outcomes

Functional outcome measures included mRS score at discharge and 3 months after surgery. Favorable and unfavorable outcomes were defined as an mRS score of 0–3 and 4–6, respectively. The mRS scores were determined by two independent clinicians (TL and ZG). CSDH recurrence was defined as radiological evidence of a subsequent increase in hematoma volume with worsening neurological symptoms and the need for reoperation. Post-operative complications, mortality, and length of hospital stay were also assessed.

### Statistical analysis

The continuous variables are presented as the means ± standard deviations, with categorical variables presented as counts and percentages. To investigate the risk factors for postoperative recurrence of CSDH, univariate and multivariate analyses were performed. Logistic regression analysis was used for univariate and multivariate analyses. Variables identified to have a *p* ≤ 0.2 in univariate analysis were entered into multivariate analysis. Differences with *p*-values <0.05 were considered statistically significant. Statistical analysis was performed using the software IBM SPSS Statistics 19.0 (IBM Corp.).

## Result

### Baseline characteristics of patients

We identified 52 consecutive patients with an average age of 83.9 years (range, 80–92 years) and a 76.9% male predominance. The majority of patients (94.3%) had a GCS score of 13 to 15, and 53.8% of patients had a score of 1 to 3 on the mRS at admission. Twenty-five (48.1%) patients had a documented history of falls or head injury in the preceding 3 months. The preexisting medical comorbidities were documented in 39 patients (75.0%), including hypertension (48.1%), cerebrovascular disease (25.0%), diabetes mellitus (23.1%), chronic obstructive pulmonary disease (15.4%), chronic kidney disease (7.7%), arrhythmia (5.8%), ischemic heart disease (5.8%), and malignancy (1.9%). Ten (19.2%) patients had taken antithrombotic medications. Limb weakness (80.8%) was the most frequent presenting symptom, followed by dizziness (40.4%), headache (28.8%), speech impairment (7.7%), incontinence (5.8%), nausea or vomiting (3.8%), seizure (3.8%), and unconsciousness (1.9%).

Seventeen (32.7%) patients suffered from bilateral CSDH; in 12 of these patients, the contralateral hematoma was <10 mm thick, and/or the neurological symptoms were not attributed to it, and unilateral SEPS was performed. Thus, a total of 57 hemispheres were operated. The mean midline shift was 7.3 ± 3.6 mm (range, 0–15 mm), and the mean CSDH maximum thickness was 23.9 ± 5.8 mm. The homogeneous type of CSDH was seen in 40.4% (23/57) of hematomas, the laminar type in 15.8% (9/57), the separated type in 17.5% (10/57), and the trabecular type in 26.3% (15/57) of hematomas. The demographic and baseline characteristics of the patients are presented in Table 1.

**Table 1:** Baseline characteristics of 52 patients with CSDH aged ≥80 years old who underwent SEPS.

**Characteristic**	**No. of patients (%)**
Mean age, yrs	83.9 ± 3.3
**Sex**	
Male	40 (76.9)
Female	12 (23.1)
**GCS score at admission**	
13–15	49 (94.3)
9–12	2 (3.8)
3–8	1 (1.9)
**mRS score at admission**	
1–3	28 (53.8)
4–5	24 (46.2)
**Recent history of falls or head injury**	25 (48.1)
**Preexisting medical comorbidities**	
All	39 (75.0)^†^
Hypertension	25 (48.1)
Cerebrovascular disease	13 (25.0)
Diabetes mellitus	12 (23.1)
Chronic obstructive pulmonary disease	8 (15.4)
Chronic kidney disease	4 (7.7)
Arrhythmia	3 (5.8)
Ischemic heart disease	3 (5.8)
Malignancy	1 (1.9)
**Previous antithrombotic medication**	10 (19.2)
**Presenting symptoms**	
Limb weakness	42 (80.8)
Dizziness	21 (40.4)
Headache	15 (28.8)
Speech impairment	4 (7.7)
Incontinence	3 (5.8)
Nausea/vomiting	2 (3.8)
Seizure	2 (3.8)
Unconsciousness	1 (1.9)
**Hematoma side**	
Right	22 (42.3)
Left	13 (25.0)
Bilateral	17 (32.7)
**Mean midline shift, mm**	7.3 ± 3.6
**Mean CSDH maximum thickness, mm**	23.9 ± 5.8
**Classification of hematoma** ^*^	
Homogeneous	23 (40.4)
Laminar	9 (15.8)
Separated	10 (17.5)
Trabecular	15 (26.3)

Values are presented as the number of patients (%) unless indicated otherwise. Mean values are presented ± SD.

GCS, Glasgow Coma Scale; mRS, Modified Rankin Scale.

† Some patients had more than one comorbidities.

* A total of 57 hematomas.

### Complications, mortality, length of stay, and functional outcome

All but one patient underwent surgery under local anesthesia alone. Postoperative complications occurred in nine patients, corresponding to an overall complication rate of 17.3% (Table 2). The new acute epidural hematoma was observed on the postoperative CT scan in two (3.8%) patients; the hemorrhage was small and neither of them required hematoma evacuation. Postoperative pneumonia was noted in six (11.5%) patients; one patient with severe pneumonia was transferred to the ICU and received a tracheotomy. Perioperative ischemic stroke occurred in two (3.8%) patients; one of them presented with contralateral malignant middle cerebral artery infarction 3 days following surgery and eventually died as a result of brain herniation. Thus, the perioperative mortality rate was 1.9% (1/52). No tension pneumocephalus developed. No patients developed wounds or intracranial infection or had epileptic seizures. In summary, the rate of serious complications was 3.8% (2/52).

**Table 2:** Results of 52 very elderly patients treated by SEPS.

**Variable**	**Total (*n* = 52)**
**Perioperative complication**	
Significant complication	2 (3.8)
All	9 (17.3)^†^
Pneumonia	6 (11.5)
Acute epidural hematoma	2 (3.8)
Ischemic stroke	2 (3.8)
**Transferred to ICU**	1 (1.9)
**Tracheostomy**	1 (1.9)
**Duration of drainage, hours**	
< 24	5 (9.6)
24–48	10 (19.2)
48–72	14 (26.9)
72–96	14 (26.9)
>96	9 (17.3)
**Poor brain expansion**	12 (23.1)
**Post-operative length of stay, days**	
Median	5
Mean	7.4 ± 6.7
Without complication	5.3 ± 2.2
With complication	17.7 ± 10.7
**Perioperative mortality**	1 (1.9)
**mRS score at discharge**	
0–3	45 (86.5)
4–6	7 (13.5)
**mRS score at 3 months**	
0–3	48 (92.3)
4–6	4 (7.7)
**Mean midline shift, mm**	1.6 ± 2.1
**Mean CSDH maximum thickness, mm**	3.2 ± 3.5
**Recurrence requiring reoperation**	5 (9.6)
**Time from initial surgery to recurrence, days**	39.6 ± 8.6

† Some patients experienced more than one complications.

**Figure 2:**
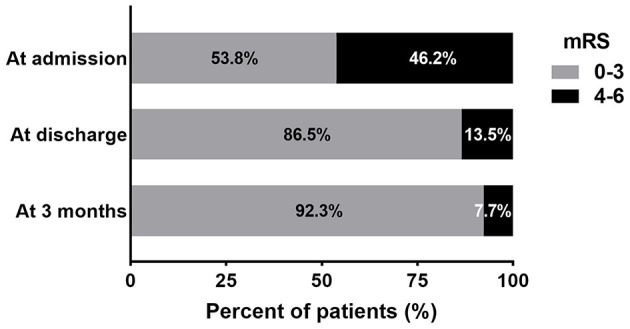
The bar diagram presents the distribution of patients according to the mRS score at admission, discharge, and 3 months following surgery.

The SEPS was removed within 96 h in 82.7% of patients. CT imaging appearance of poor brain expansion was seen in 12 (23.1%) patients. The mean maximum thickness of hematoma was significantly reduced after SEPS (23.9 ± 5.8 mm preoperative vs. 3.2 ± 3.5 mm at discharge, *p* < 0.001). The mean midline shift at discharge was 1.6 ± 2.1 mm (range, 0–7 mm). The SEPS resulted in significant midline shift improvement (*p* < 0.001). The median and mean postoperative length of stay were 5 and 7.4 ± 6.7 days (range, 2–43 days), respectively. The average postoperative length of stay was significantly longer for patients who developed complications (17.7 ± 10.7 days vs. 5.3 ± 2.2 days; *p* < 0.001). A total of 45 (86.5%) patients had a favorable outcome (mRS score 0–3) at discharge; 48/52 (92.3%) achieved this at 3 months follow-up (Figure 2).

### Recurrence of CSDH and risk factors associated

Five patients (9.6%) experienced CSDH recurrence and required reoperation; all of them underwent repeat SEPS. The average interval between the initial operation and reoperation was 39.6 ± 8.6 days (range, 30–49 days). The univariate analysis revealed that previous antithrombotic medication (*p* = 0.032) and CT presence of poor brain expansion (*p* = 0.012) were potential risk factors for the recurrence of CSDH. Multivariate regression analysis indicated that poor brain expansion was the only significant predictor for CSDH recurrence (*p* = 0.034).

## Discussion

### The functional outcome, mortality, and complication rate in very elderly patients with CSDH

The incidence of CSDH is increasing, especially in elderly patients as life expectancy increases. The management of symptomatic CSDH without new-onset morbidity can be challenging in elderly patients due to their preexisting illnesses and advanced age. Although surgical drainage is minimally invasive and is the predominant treatment of CSDH, the procedure still carries high morbidity and mortality rates for the geriatric population (19). In 2013, Stippler et al. (20) reported 21 patients older than 90 years and found that the outcome is poor, with high inpatient mortality rates (24%) regardless of whether they received surgical treatment or not. There has been controversy over which surgical technique is the most effective; however, there is no conclusive evidence for each technique yet.

In this study, we present a series of patients aged ≥80 years old who underwent SEPS with subdural thrombolysis. Similarities and differences exist between our results and those reported for BHD. The preexisting comorbidities were observed in approximately 80% of patients in our series. Nonetheless, their clinical status progressively improved after surgery and continued to ameliorate until the last follow-up. Over 90% of patients had an mRS of 0–3 at 3 months following surgery. This result should be viewed in the context of patients' mean age of 83.9 years and their high prevalence of comorbidities.

A summary of previous studies on BHD for elderly patients older than 80 years is presented in Table 3. In the literature, the mortality rate during hospitalization or at 30 days for very elderly patients with CSDH after BHD ranges from 3.6 to 7.1% (2, 3, 7, 8, 21–25). In the present study, the perioperative mortality rate was 1.9%. The one death was not directly related to surgery. This patient with a severe preexisting condition suffered from massive cerebral infarction and died of subsequent serious herniation. The low mortality and the proportion of patients who achieved good functional outcomes were not expected. Ou et al. (7) also reported a similar proportion of patients (94.3%) achieving good outcomes with a mortality rate of 4.5%.

**Table 3:** Summary of prior studies on burr-hole drainage for CSDH in very elderly patients older than 80 years.

**Author**	**Type of anesthesia**	**No. of pts**	**Age group (yrs)**	**Complication, *n* (%)**	**Mortality, *n* (%)**	**Length of hospital stay (days)**	**Good outcome, *n* (%)**	**Recurrence requiring reoperation, *n* (%)**
Borger et al. (21)	General anesthesia	53	≥85	5 (9.4)	2 (3.8)	NR	27 (51.0), mRS 0–2 at discharge	4 (7.5)
Cheng et al. (22)	General anesthesia	55	≥85	5 (9.1)	3 (5.5)	NR	39 (71.0), GOS 4–5 at discharge	2 (3.6)
Lee et al. (23)	General/local anesthesia	70	≥90	8 (11.4)	5 (7.1)	16.9 ± 13.2	NR	3 (4.3)
Munoz-Bendix et al. (8)	General anesthesia	56	≥85	5 (8.9)	2 (3.6)	9.8	50 (89.3), GOS 4–5 at 1 month	14 (25.0)
Bartek et al. (24)	Local anesthesia and sedation	75	≥90	3 (4.1)	3 (4.0), at 30-day	NR	NR	8 (10.7%)
Dobran et al. (2)	Local anesthesia	25	≥90	1 (4.0)	1 (4.0)	NR	21 (84.0), Markwalder 0–1 at 1 month	5 (20.0)
Ou et al. (7)	NR	158	≥80	15 (9.5)	7 (4.5)	8.1 ± 3.6	149 (94.3), mRS 0–3 at 3 months	2 (1.3)
Watanabe et al. (25)	Local anesthesia and sedation	73	≥80	4 (5.5)	4 (5.5)	NR	NR	11 (15.1)
Greuter et al. (3)	NR	104	≥80	39 (37.5)	4 (3.8), at 30-day	7 (median)	81 (77.9), mRS 0–3 at 6 weeks	11 (10.6)

NR, not reported; mRS, Modified Rankin Scale; GOS, Glasgow Outcome Scale.

Regarding complication rates, a review of the literature reveals that postoperative complications develop in 4.0 to 37.5% of cases undergoing BHD (2, 3, 7, 8, 21–25). In the present series, the overall complication rate was 17.3%. Nevertheless, severe complications were noted in two patients (3.8%). The most common complication was pneumonia and seen in 11.5% of patients. The majority of patients did not experience surgery-related complications, and the median postoperative length of stay was <1 week. When elderly patients experience a complication, it more than trebles their postoperative length of stay (17.7 vs. 5.3 days, *p* < 0.001). Few data regarding hospitalization time are available in the literature. These statistics are useful for preoperative counseling for elderly patients with CSDH and their families.

### The recurrence rate after SEPS and subdural thrombolysis is comparable to that in patients treated by BHD in the literature

In the literature, rates of recurrence requiring reoperation for elderly patients treated with BHD vary between 1.3 and 25.0% (2, 3, 7, 8, 21–25). Our observed recurrence rate of 9.6% following SEPS was, therefore, consistent with these previously reported rates and represented comparatively low incidence. These results supported that SEPS with thrombolysis is a good option with an efficacy comparable to BHD.

Results from other reports demonstrated increased recurrence rates following SEPS (10, 12). The additional urokinase thrombolysis in the subdural space might account for the decrease in recurrence in our series. Prolonged postoperative widening of the subdural cavity, which results from residual hematoma or massive air collection, tends to promote CSDH recurrence by causing impaired adhesion between the inner and outer neomembranes (26–29). Although SEPS was able to remove a large amount of subdural hematoma, efficient evacuation of residual hematoma benefited from repeated urokinase thrombolysis. Recent clinical trials confirmed that intraventricular thrombolysis can safely remove blood clots *via* a routine external ventricular drain in patients with intraventricular hemorrhage (30, 31). Similarly, gentle subdural thrombolysis could promote residual hematoma evacuation and prevent subsequent hematoma recurrence. Some previous studies have described the use of urokinase in the subdural space (15–17). Subdural urokinase administration was found to be safe and effective for improving drainage. Ou et al. (16) designated this strategy as exhaustive drainage.

In addition, subdural thrombolysis could keep the system from becoming blocked or from inadequate drainage. Flint et al. (13) reported that some modifications implemented to minimize intraprocedural clotting of SEPS have significantly decreased the recurrence rate. In fact, these incremental modifications of the SEPS technique parallel our theory of subdural thrombolysis, although each takes a different path toward the very same goal. In the present series, for more than 70% of patients, the drainage continued for over 48 h. The extended duration of drainage also leads to less residual hematoma and a greater extent of cerebral re-expansion resulting in the narrowing of the subdural space.

### Advantages of SEPS compared with conventional BHD

A SEPS is technically less complex and invasive than BHD, resulting in a reduction of the surgical duration. In some neurosurgical centers, SEPS was even performed at the bedside in a monitored setting and therefore avoided the utilization of expensive and time-limited operating room resources. Safain et al. (9) found that the safety and effectiveness of bedside SEPS were similar to that of BHD or small craniotomy.

BHD is usually performed under general anesthesia (32, 33). However, CSDH is more common among elderly patients who tend to have multiple comorbidities and, thus, are at increased risk for general anesthesia (34–36). Although local anesthesia has been reported during BHD by some authors (37, 38), patients may still become anxious and experience discomfort during the procedure. A prospective randomized study suggested that conscious sedation with local anesthesia is safe and effective for BHD (39). Significantly fewer postoperative complications and shorter lengths of hospital stay were observed in the local anesthesia group when compared with the general anesthesia group. Due to its technically easy and minimally invasive nature, SEPS can be performed with local anesthesia alone. In our study, preexisting comorbidities were documented in 75% of the aged patients who were deemed too fragile to undergo general anesthesia and at high risk of perioperative complications. With this in mind, almost all procedures were completed under local anesthesia. This technique enables immediate neurological improvement and favorable outcomes. In addition, due to its technical advantages in terms of minimal invasion and easier hematoma evacuation compared with standard BHD, SEPS can also be advocated for young patients who might have contraindications to general anesthesia and might be best placed for drainage under local anesthesia.

Unlike the BHD technique, SEPS provides an airtight seal without leaving a catheter in the subdural space, thereby minimizing the risk of intraoperative and postoperative pneumocephalus. In our series, no patients experienced pneumocephalus. In contrast, massive subdural air collection has been identified as one of the complications following BHD and found to be associated with CSDH recurrence (26, 29). The air bubble may contribute to poor brain expansion and maintain a potential subdural space for the reaccumulation of blood and fluid.

The subdural catheter which is used for intraoperative irrigation and postoperative drainage in BHD is positioned between the dura and the cortex, in direct contact with the cortical surface, bridging veins, and hematoma membranes, which may lead to seizures, brain laceration, and bleeding from injury of these structures. In SEPS, the absence of a subdural catheter is considered safer and might limit seizures by avoiding cortical irritation and potential epileptogenic effects. Postoperative seizures and intraparenchymal hemorrhage were not encountered in the present study.

Middle meningeal artery (MMA) embolization, which could also be performed under local anesthesia, has emerged as an endovascular treatment option for CSDH. The goal of MMA embolization is to control bleeding from the CSDH membrane and eventually enhance the spontaneous resolution of the hematoma. MMA embolization has been employed as the sole management for mild symptomatic CSDH or as a preoperative or postoperative adjunct to surgical evacuation of CSDH with the intention of reducing postoperative recurrence (40–42). Multiple randomized clinical trials are ongoing to demonstrate the continued safety and efficacy of this technique.

### Limitations

There are some limitations that should be mentioned. This is a retrospective study with a small number of cases assessed. However, this is one of the few reports describing the SEPS technique for very elderly patients, and it could serve as a valuable starting point to raise specific questions regarding optimal surgical treatment for this subpopulation. There was no control cohort in our study since SEPS had become established at our institution as a first-line treatment for symptomatic CSDH in the last decade. A review of the literature enables a comparison of complications, mortality, and recurrence rates between SEPS and BHD. Further prospective multicenter studies are necessary to compare these two different surgical techniques directly.

## Conclusion

As an exhaustive drainage strategy, SEPS followed with thrombolysis is safe and effective with favorable outcomes in elderly patients with CSDH aged ≥80 years old. It is technically a less complex and invasive procedure with similar complications, mortality, and recurrence rates when compared with BHD in the current literature.

## Data availability statement

The raw data supporting the conclusions of this article will be made available by the authors, without undue reservation.

## Ethics statement

The studies involving human participants were reviewed and approved by Ethics Committee of Longyan First Hospital, Fujian Medical University. The patients/participants provided their written informed consent to participate in this study.

## Author contributions

ZL conceptualized and designed the study and supervised the study. TL, ZG, JZho, XL, QR, and XC collected the data. TL, ZG, DG, and JZhe analyzed and interpreted the data. TL and ZG drafted the article. TL, ZG, DG, JZhe, FL, YL, and ZL critically revised the article. All authors contributed to the article and approved the submitted version.
